# Comprehensive rehabilitation and resocialization in mental health care after surgery of head and neck tumors

**DOI:** 10.1192/j.eurpsy.2022.1581

**Published:** 2022-09-01

**Authors:** M. Magomed-Eminov, O. Orlova, D. Uklonskaya, D. Reshetov, Y. Zborovskaya, S. Matveeva, E. Romanova, M. Fedina

**Affiliations:** 1 Lomonosov Moscow State University, Department Of Psychological Help And Resocialization, Moscow, Russian Federation; 2 The National Medical Research Center for Otorhinolaryngology of the FMBA of Russia, Phoniatrics Department, Moscow, Russian Federation; 3 Central Clinical Hospital “RZD-Medicine”, Oncology Department №2 (head And Neck Tumors), Moscow, Russian Federation

**Keywords:** mental health, rehabilitation, head and neck tumors, resocialization

## Abstract

**Introduction:**

There is increasing number of head and neck tumors. Modern medical technologies allow to save lives, but lead to cosmetic and functional defects. DSM-V clarifies that “life threatening illness or debilitating medical condition is not necessarily considered a traumatic event”. However, cancer diagnosis and treatment influence on mental health. Patients after surgery of head and neck tumors need special rehabilitation, because of loss or impairment of speech function. This significantly reduces communicative potential, changes social status, reduces rehabilitation potential.

**Objectives:**

During 6 years we have conducted studies to improve methods of psychological and pedagogical diagnostics, optimize speech therapy and psychological support for increasing effectiveness of speech rehabilitation.

**Methods:**

Speech rehabilitation was carried out with correctional-pedagogical technologies and psychological support. We used Achieving Tendency Scale, Questionnaire measures of Affiliative Tendency and Sensitivity to Rejection, narrative interview, Scale of speech utterance implementation, rehabilitation potential evaluation, general condition assessment by ECOG and Karnovsky, auditory assessment. Speech therapy included methods for eliminating dysphagia, normalizing speech breathing, improving utterance realization. Psychological support kept meaning-narrative approach.

**Results:**

Number of patients without difficulties or with slight difficulties in speech utterance implementation increased by 57.1%. Number of patients with high rehabilitation potential increased by 48.8%.

**Conclusions:**

Psychological and pedagogical rehabilitation and resocialization after surgery of head and neck tumors has positive effect on mental health of patients, forms special rehabilitation motivation, helps to avoid disability and to transform life in new conditions.

**Disclosure:**

No significant relationships.

